# Translatable *in‐vivo* investigation of effects of progressive hypoxia in pregnancy on fetal cardiac structure and function in sheep

**DOI:** 10.1002/uog.29306

**Published:** 2025-08-18

**Authors:** O. V. Patey, K. J. Botting‐Lawford, Y. Niu, L. Zhang, J. Ma, S. G. Ford, W. Tong, C. M. Coutinho, B. Thilaganathan, D. A. Giussani

**Affiliations:** ^1^ Department of Physiology, Development and Neuroscience University of Cambridge Cambridge UK; ^2^ Fetal Medicine Unit St George's University Hospitals NHS Foundation Trust London UK; ^3^ Brompton Centre for Fetal Cardiology, Royal Brompton and Harefield Hospitals, Guy's and St Thomas' NHS Foundation Trust London UK; ^4^ Department of Aerospace Physiology Fourth Military Medical University Xi'an China; ^5^ Department of Gynecology and Obstetrics, Ribeirão Preto Medical School University of São Paulo Ribeirão Preto Brazil

**Keywords:** fetal echocardiography, fetal heart, fetal hypoxia, left ventricular twist, speckle tracking, tissue Doppler imaging

## Abstract

**Objective:**

Human fetal growth restriction (FGR) is associated with cardiac dysfunction. However, it remains unclear whether alterations in the fetal heart in human pregnancy affected by FGR are a consequence of chronic fetal hypoxia. In this study, we used novel *in‐vivo* ultrasound imaging modalities in pregnant sheep to evaluate fetal cardiac responses to progressive hypoxia in late gestation.

**Methods:**

This was a prospective study of data collected between July 2019 and March 2022 from 42 Welsh mountain ewes, each carrying a single fetus. Seventeen ewes were exposed to hypoxia in isobaric chambers (10% oxygen (O_2_)) from 105 days of gestational age (dGA; with term being approximately 147 days) for 11 ± 1 days (H1, *n* = 5), 17 ± 1 days (H2, *n* = 6) or 32 ± 2 days (H3, *n* = 6). An ultrasound system with vendor‐specific software was used to determine fetal left ventricular (LV) and right ventricular (RV) geometry and function. Outcomes were compared with the remaining 25 gestational age‐matched ovine normoxic control pregnancies at 117 ± 1 (N1, *n* = 13), 124 ± 1 (N2, *n* = 5) and 133 ± 3 (N3, *n* = 7) dGA.

**Results:**

Compared to controls with expected fetal RV dominance in late gestation, hypoxic fetal sheep showed LV dominance with increasing duration of hypoxia, including statistically significant increases in mean ± SD LV/RV end‐diastolic area ratio (N1, 1.1 ± 0.2 *vs* H1, 1.3 ± 0.1; N2, 1.2 ± 0.4 *vs* H2, 1.7 ± 0.5; N3, 0.9 ± 0.1 *vs* H3, 1.4 ± 0.2), LV sphericity index (N3, 0.44 ± 0.04 *vs* H3, 0.54 ± 0.11) and LV/RV cardiac output ratio (N1, 0.55 ± 0.18 *vs* H1, 1.00 ± 0.31; N2, 0.80 ± 0.10 *vs* H2, 1.62 ± 0.59; N3, 0.74 ± 0.23 *vs* H3, 1.50 ± 0.58). The mean ± SD LV myocardial performance index was significantly greater in the hypoxic groups, signifying global myocardial dysfunction (N1, 0.45 ± 0.07 *vs* H1, 0.64 ± 0.07; N2, 0.40 ± 0.05 *vs* H2, 0.66 ± 0.07; N3, 0.36 ± 0.07 *vs* H3, 0.60 ± 0.10). While LV apical radial strain and LV apical systolic rotation were initially increased after 17 days of hypoxia, these indices were significantly reduced after 32 days of hypoxia (median LV apical radial strain: N1, 27% (interquartile range (IQR), 26–28%) *vs* H1, 24% (IQR, 21–26%); N2, 24% (IQR, 22–25%) *vs* H2, 70% (IQR, 66–79%); N3, 51% (IQR, 48–54%) *vs* H3, 29% (IQR, 27–32%); mean ± SD LV apical systolic rotation: N1, 7 ± 5° *vs* H1, 5 ± 3°; N2, 9 ± 1° *vs* H2, 14 ± 2°; N3, 15 ± 2° *vs* H3, 7 ± 2°). Hypoxic fetuses showed biventricular hypertrophy and evidence of biventricular diastolic dysfunction, with significant LV impairment presenting after 11 days of hypoxia (mean ± SD LV isovolumetric relaxation time (IVRT′) normalized by cardiac cycle (cc) length: N1, 0.10 ± 0.02 ms *vs* H1, 0.15 ± 0.02 ms; N2, 0.09 ± 0.01 ms *vs* H2, 0.14 ± 0.02 ms; N3, 0.08 ± 0.03 ms *vs* H3, 0.12 ± 0.02 ms), preceding RV impairment after 32 days of chronic hypoxia (mean ± SD RV‐IVRT′ normalized by cc length: N3, 0.08 ± 0.03 ms *vs* H3, 0.14 ± 0.03 ms).

**Conclusions:**

Progressive fetal hypoxia in sheep leads to profound changes in fetal cardiac structure and function, resulting from a switch to LV dominance triggered by fetal brain sparing. The findings also indicate that fetal cardiac compensatory reserves become exhausted with progressive hypoxia. © 2025 The Author(s). *Ultrasound in Obstetrics & Gynecology* published by John Wiley & Sons Ltd on behalf of International Society of Ultrasound in Obstetrics and Gynecology.

## INTRODUCTION

Human pregnancy complicated by fetal growth restriction (FGR) can lead to fetal cardiac remodeling and dysfunction, and these alterations may persist postnatally, increasing the cardiovascular risk in later life^1^, ^2^. Therefore, there is interest in understanding the mechanisms driving these associations to improve the surveillance and treatment of those affected by FGR.

It is generally assumed that fetal cardiac remodeling in FGR pregnancies results from chronic fetal hypoxia rather than fetal undernutrition, triggered via the well‐known fetal brain sparing circulatory response to impaired fetal oxygenation^1^. It has been established that fetal hypoxia results in local dilatation of the cerebral blood vessels as well as triggering a carotid chemoreflex, increasing sympathetic outflow that promotes constriction of the peripheral vasculature^3^, ^4^, ^5^. The preferential blood streaming across the foramen ovale into the left side of the fetal heart directs blood flow towards the fetal brain, resulting in left ventricular (LV) volume overload^6^. While fetal cerebral vasodilatation decreases LV afterload, fetal peripheral vasoconstriction increases placental vascular resistance, elevating fetal right ventricular (RV) afterload^1^, ^3^, ^7^. Cardiac loading leads to remodeling, but the nature of the remodeling differs depending on whether there is excess preload or afterload^8^. Chronic volume loading promotes chamber dilatation and eccentric hypertrophy, whereas pressure overload results in concentric hypertrophy, reducing chamber size^8^, ^9^. Consequently, chronic fetal hypoxia is likely to induce remodeling of the fetal heart; however, this hypothesis has not been tested systematically, especially with respect to comparison of different durations of fetal hypoxic exposure.

Sheep and humans share similar milestones in fetal cardiovascular development^1^, ^10^; therefore, studies using pregnant sheep are of high human translational value. In the present study, we applied advanced *in‐vivo* ultrasound modalities to pregnant sheep exposed to different durations of chronic hypoxia in late gestation using a bespoke hypoxic chamber system to determine the effect on fetal cardiac adaptation. This study tested the hypothesis that progressive chronic hypoxia differentially affects cardiac structure and function in the sheep fetus.

## METHODS

### Induction of chronic hypoxic pregnancy and experimental groups

All procedures were performed at The Barcroft Centre of the University of Cambridge under the UK Animals (Scientific Procedures) Act 1986, and were approved by the Ethical Review Board of the University of Cambridge (No: PP6755721). The experimental design was conducted following the ARRIVE and PREPARE guidelines.

**Figure 1:**
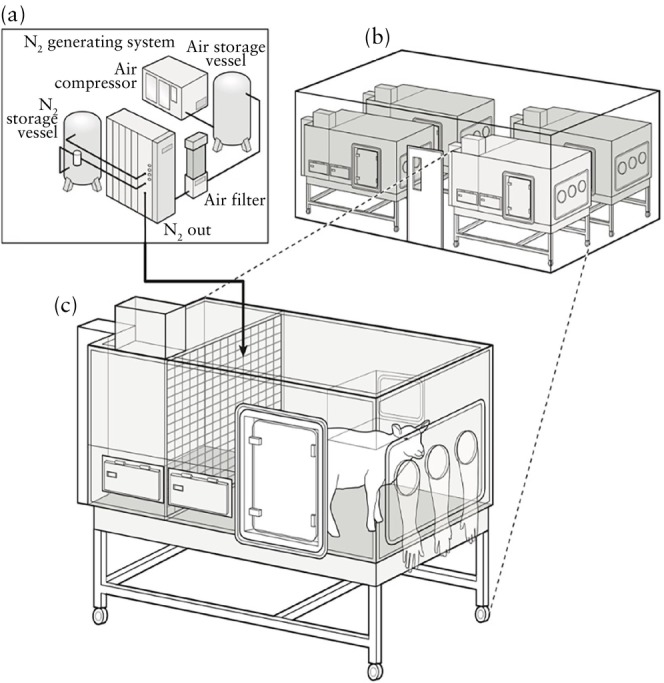
Bespoke isobaric hypoxic chambers. (a–c) Specially designed nitrogen (N_2_)‐generating system (a) supplied variable amounts of compressed air and N_2_ to bespoke isobaric hypoxic chambers housed in hypoxic chamber laboratory (b,c). Each chamber had electronic humidity cool stream injection system, drinking bowl on continuous water supply and rotating food compartment for controlled food intake. Waste was disposed of via sealed waste pipe. Chambers were transparent, allowing pregnant sheep to see each other. Figure is reproduced with permission from Brain *et al*.^11^.

This was a prospective study of data collected between July 2019 and March 2022 from 42 Welsh mountain ewes carrying a single fetus, as determined by ultrasound (Toshiba Medical Systems Europe, Zoetermeer, The Netherlands) at 80 days of gestational age (dGA; with term at approximately 147 days). Pregnancies were assigned randomly to either chronic normoxia (N, *n* = 25) or chronic hypoxia (H, *n* = 17) using a random number generator at 105 dGA. Chronic hypoxia was induced via bespoke isobaric hypoxic chambers housed at The Barcroft Centre (Figure 1) using established procedures^5^, ^11^, ^12^, ^13^. Briefly, the ewes were fed a bespoke concentrate and hay‐pellet maintenance diet, so that food intake could be monitored (Cambridge ewe diet: 40 g nuts/kg and 3 g hay/kg; Manor Farm Feeds Ltd, Oakham, UK)^11^. Each hypoxic chamber was supplied with controlled volumes of nitrogen and air generated via a bespoke hypoxia generating system (Domnick Hunter Gas Generation, Gateshead, UK)^5^, ^11^, ^12^, ^13^. The inspirate was passed via filters and silencers to reduce noise, providing a quiet environment for the animal inside each chamber. The pregnant ewes were in view of other ewes. The volume of gas in each chamber was replaced a minimum of 12 times per hour. All chambers were equipped with humidifiers (1100‐03239 HS‐SINF Masalles, Barcelona, Spain), and ambient partial pressure of oxygen (pO_2_), partial pressure of carbon dioxide, humidity and temperature within each chamber were monitored via sensors and values recorded using the Trends Building Management System of the University of Cambridge. In this way, the pO_2_ within each chamber could be controlled with precision over long periods.

**Figure 2:**
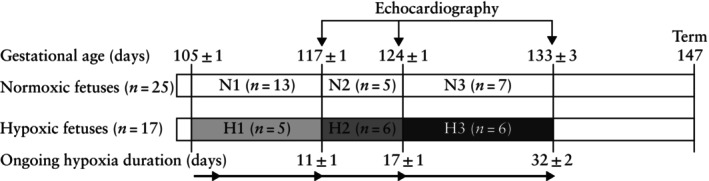
Overview of experimental timeline. At 105 days of gestation (dGA; term is at 147 dGA), ewes were randomly assigned to either normoxic or hypoxic pregnancy groups. The first group of ewes subjected to chronic hypoxia was exposed to 10% oxygen (O_2_) for 11 ± 1 days (H1, *n* = 5), with echocardiography performed at 117 ± 1 dGA. The second group of ewes was exposed to 10% O_2_ for 17 ± 1 days (H2, *n* = 6), with echocardiography performed at 124 ± 1 dGA. The third group of ewes was exposed to 10% O_2_ for 32 ± 2 days (H3, *n* = 6), with echocardiography performed at 133 ± 3 dGA. Echocardiography was also performed in gestational age‐matched control normoxic pregnancies at 117 ± 1 dGA (N1, *n* = 13), at 124 ± 1 dGA (N2, *n* = 5) and at 133 ± 3 dGA (N3, *n* = 7).

Ewes randomly assigned to hypoxic pregnancy at 105 dGA were gradually subjected to hypoxia, reaching 10 ± 1% inspired oxygen over 24 h. This protocol resulted in a fetal mean pO_2_ in arterial blood of 11.5 ± 0.6 mmHg, measured in the descending aorta of chronically catheterized fetal sheep undergoing the same experimental procedure^5^, ^14^, ^15^. This level of chronic fetal hypoxia is similar to the levels of pO_2_ measured in the umbilical cord of human fetuses from pregnancies complicated by FGR and chronic fetal hypoxia^16^, ^17^. The first group of ewes subjected to chronic hypoxic pregnancy were exposed to 10% oxygen (O_2_) chronic hypoxia from 105 dGA for 11 ± 1 days (H1, *n* = 5). Echocardiography was performed in H1 pregnancies at 117 ± 1 dGA. The second group of ewes was exposed to 10% O_2_ chronic hypoxia for 17 ± 1 days (H2, *n* = 6). Echocardiography was performed in H2 pregnancies at 124 ± 1 dGA. The third group of ewes was exposed to 10% O_2_ chronic hypoxia for 32 ± 2 days (H3, *n* = 6). Echocardiography was performed in H3 pregnancies at 133 ± 3 dGA. The remaining 25 pregnancies served as gestational age‐matched normoxic controls. Echocardiography was performed in N1 (*n* = 13) at 117 ± 1 dGA, in N2 (*n* = 5) at 124 ± 1 dGA and in N3 (*n* = 7) at 133 ± 3 dGA (Figure 2). Longitudinal assessment of echocardiographic indices of cardiac structure and function could not be performed at all three study ages in some control and hypoxic pregnancy cases because tissues had to be harvested at postmortem for assessment of changes in molecular biology as part of other studies. The normoxic control sheep were kept in a barn in floor pens with the same floor area as that of the hypoxic chambers and fed the same bespoke maintenance diet as the hypoxic ewes.

### Fetal echocardiography

Fetal heart assessment was conducted in the conscious animal via maternal transabdominal ultrasound examination without any anesthesia and after shaving the abdominal hair. To avoid any effect of maternal stress on fetal echocardiographic assessment, ewes maintained at The Barcroft Centre are conditioned for at least 8 weeks to habituate them to ultrasound scanning. To confirm lack of maternal stress, the maternal heart rate was measured during all echocardiographic procedures. One investigator (O.V.P.) performed fetal echocardiography using a Vivid iq ultrasound system with a linear M5S transducer (GE Healthcare, Zipf, Austria). The ewes in the hypoxic group were removed from the hypoxic chambers during the fetal echocardiographic examination but remained under the same hypoxic conditions, maintained by delivering the hypoxic inspirate via a respiratory hood. All ewes were in a semi‐supine position, with their backs supported by one technician (S.G.F.) during the scan.

Fetal LV and RV geometry and function were determined. Fetal M‐mode, B‐mode and pulsed‐wave (PW) Doppler measurements, as well as PW tissue Doppler imaging (TDI) curves and two‐dimensional images for speckle‐tracking echocardiography (STE) analysis, were obtained and recorded. M‐mode ultrasound was used for the assessment of LV radial function (measured by the shortening fraction (SF), in %) and ventricular longitudinal motion (measured by mitral (MAPSE), tricuspid and septal (SAPSE) annular plane systolic excursion (in mm)). B‐mode imaging was performed for measurement of the ventricular chambers in end‐systole and end‐diastole, as well as measurement of aortic valve (AV, in mm) and pulmonary valve (PV, in mm) end‐systolic dimensions, mitral valve (MV, in mm) and tricuspid valve (TV, in mm) end‐diastolic dimensions, ventricular wall and interventricular septum (IVS) thickness (in mm) in end‐diastole, and both LV and RV sphericity indices (SI). Relative wall thickness (RWT) was estimated using the equation: RWT = (wall thickness)^2^/ventricular end‐diastolic dimension (EDD). EDD (in mm) and end‐systolic dimension (ESD, in mm) were the ventricular width or transverse diameter measured at the basal level in the four‐chamber view in diastole and systole, respectively. SI was calculated by dividing ventricular EDD by end‐diastolic length (EDL, in mm) for each ventricle^18^. LV‐SF (%) was calculated as SF = ((EDD − ESD)/EDD) × 100. By manually tracing the RV endocardium in diastole and systole in the four‐chamber view, the end‐diastolic area (EDA, in mm^2^) and end‐systolic area (ESA, in mm^2^) were derived, and the RV fractional area change (FAC, in %) was estimated as FAC = ((EDA − ESA)/EDA) × 100. The PW Doppler technique was used to obtain Doppler signals from the inflow (early (E) and late (A) diastolic velocities (in cm/s) and the E/A ratio) and outflow tracts for evaluation of diastolic and systolic function, respectively, together with calculation of LV and RV stroke volume (in mL) and cardiac output (CO, in mL/min). CO for both ventricles was calculated as CO = outflow tract dimension × velocity time integral × fetal heart rate. The PW‐TDI technique was applied for both ventricles to derive cardiac indices of myocardial motion in systole (peak systolic myocardial velocities (cm/s)) and peak diastolic myocardial velocities (E′ and A′ (in cm/s) and E′/A′ ratio) and myocardial time intervals, such as isovolumetric contraction time (IVCT′, in ms), ejection time (ET′, in ms), isovolumetric relaxation time (IVRT′, in ms) and relaxation time (RT′, in ms). LV and RV myocardial performance index (MPI′) was calculated as MPI′ = (IVCT′ + IVRT′)/ET′.

STE was used to derive global myocardial deformation indices (LV and RV longitudinal peak systolic strain and strain rate, and LV circumferential, radial and rotational peak systolic strain and strain rate at the basal and apical levels) with a frame rate of > 100 frames per s. With the dummy electrocardiogram (Lionheart 2 BIO‐TEK® Multiparameter Simulator; BIO‐TEK Instruments, Winooski, VT, USA) set at 60 bpm, the system was able to capture two consecutive fetal cardiac cycles or at least one whole fetal cardiac cycle with a real fetal heart rate of 120–150 bpm. Several digital clips were obtained and then transferred to the dedicated software (EchoPAC version 112; GE Healthcare) for further analysis. LV net twist was calculated as the difference between the peak basal rotation and the peak apical rotation. LV torsion was estimated as the net twist divided by the LV‐EDL. STE analysis of rotational direction was adjusted with respect to the fetal orientation on the ultrasound screen^19^. The number of fetuses that presented with post‐systolic shortening (PSS) was assessed from the LV longitudinal strain STE curves^20^. Myocardial time intervals were normalized by cardiac cycle length, adjusting for variation in heart rate. The normalized values were used for comparisons^21^, ^22^, ^23^, ^24^, ^25^. ‘Minus’ or ‘plus’ signs before strain values indicate lengthening (for longitudinal and circumferential strain) or shortening (for radial strain) of the myocardium, respectively; these signs were ignored when comparisons between strain values were made^23^, ^24^, ^25^. All measurements were performed in a single beat according to standardized protocols described for fetal echocardiography^19^, ^23^, ^24^, ^25^.

### Intra‐ and interobserver reproducibility

The same observer (O.V.P.) repeated all PW‐TDI and STE measurements in 10 randomly chosen fetal echocardiograms in a different cardiac cycle using the EchoPAC software. A second observer (C.M.C.) repeated these measurements in a different cardiac cycle in the same 10 fetal echocardiograms but in a randomly chosen order. The observers were blinded to each other's measurements.

### Statistical analysis

The power calculations were specifically designed to compare six unpaired groups, investigating the main effects of treatment (normoxia *vs* hypoxia) and gestational age. Myocardial deformation parameters obtained by STE are considered the most sensitive cardiac indices, as they reflect the earliest ischemic alterations in the myocardium compared to other strain measurements, such as circumferential and radial. This is because the longitudinal myocardial fibers are situated further away from the coronary circulation^8^. Moreover, systolic strain rate has been reported to be a more robust measure of contractility that is less influenced by changes in cardiac load and structure^26^. For these reasons, strain rate was considered a primary outcome, and a sample of 30 pregnancies was calculated to be able to detect a rate of change (length/s) difference of 0.21 (equivalent to 10% of the mean, 2.05), with a power of 85%, significance level of 5% and assuming a standard deviation of 0.28^25^. To allow for possible confounding factors including experimental failure and to ensure sufficient power, the sample size was increased to 42 pregnancies. Statistical analysis was performed using SPSS version 22.0 (SPSS Inc., Chicago, IL, USA). Both the Shapiro–Wilk and Kolmogorov–Smirnov tests were performed to determine the normality of the data distribution. Normally distributed data were analyzed using ANOVA with Tukey's Honest Significant Difference post‐hoc test, and the Kruskal–Wallis test was used for non‐normally distributed data. Differences between groups were considered statistically significant if the two‐tailed *P*‐values were < 0.05. Linear regression analysis was used to explore gender‐related differences in measured cardiac outcomes. For the intra‐ and interobserver repeatability of PW‐TDI and STE indices, the Bland–Altman analysis (including limits of agreement and Pitman's test for variance differences) was used and intraclass correlation coefficients (ICCs) were calculated to assess the intra‐ and interobserver repeatability of PW‐TDI and STE indices.

## RESULTS

There were no significant differences between the normoxic and hypoxic groups in mean ± SD gestational age at echocardiography (N1, 117 ± 1 *vs* H1, 117 ± 1 days; N2, 124 ± 1 *vs* H2, 124 ± 1 days; N3, 133 ± 3 *vs* H3, 133 ± 3 days) or in fetal sex (% male) (N1, 46% *vs* H1, 50%; N2, 45% *vs* H2, 50%; N3, 39% *vs* H3, 50%) at all timepoints of assessment. There were no gender associations with cardiac parameters in any of the fetal groups.

### Cardiac alterations at 11 days of hypoxia

**Figure 3:**
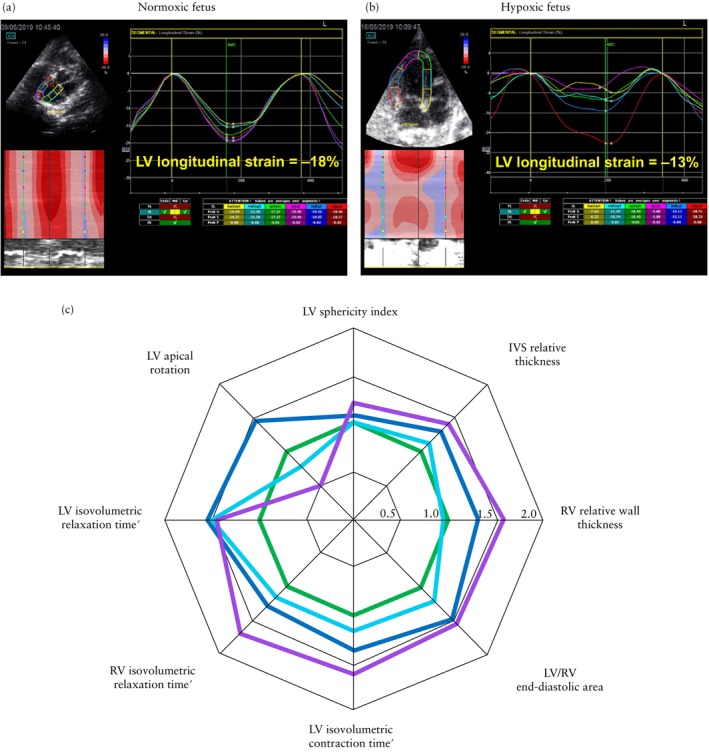
Summary of selected altered geometrical and functional cardiac parameters in chronically hypoxic sheep fetuses compared to normoxic group. Speckle‐tracking myocardial deformation curves showing systolic peaks of regional and global left ventricular (LV) longitudinal strain in normoxic sheep fetus at 124 days of gestation (a) and gestational age‐matched hypoxic fetus after 17 days of chronic hypoxia (b). (c) Spiderweb plot showing significant percentage alterations from mean normoxic values for indices of cardiac geometry and function in chronically hypoxic sheep fetuses after 11 days (light blue, H1 (*n* = 5)), after 17 days (dark blue, H2 (*n* = 6)) and after 32 days (purple, H3 (*n* = 6)) of hypoxic exposure compared to gestational age‐matched normoxic controls (green, represented as 100% (*n* = 25)). Myocardial time intervals were normalized by cardiac cycle length, adjusting for variation in heart rate. IVS, interventricular septum; RV, right ventricular.

**Figure 4:**
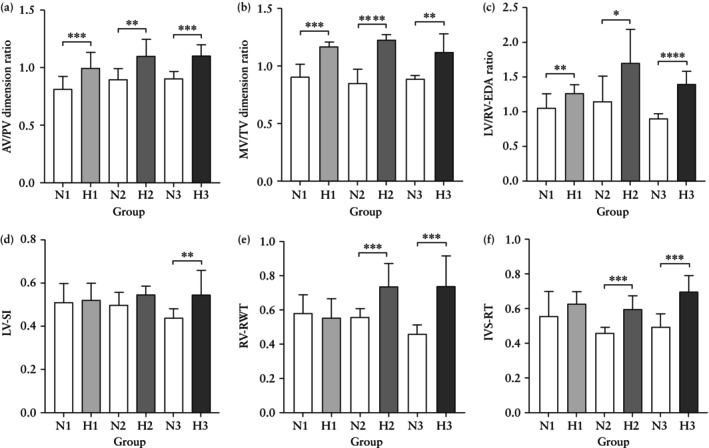
(a–f) Cardiac geometry in normoxic and chronically hypoxic sheep fetuses. Data are shown as column plots with error bars representing mean ± SD for alterations in cardiac geometry in chronically hypoxic sheep fetuses after 11 days (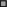, H1), after 17 days (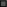, H2) and after 32 days (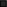, H3) of chronic hypoxia compared to gestational age‐matched normoxic controls (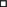, N1; N2; N3). Significant differences are for comparison of normoxic *vs* hypoxic groups: *, *P* < 0.05; **, *P* < 0.01; ***, *P* < 0.001; ****, *P* < 0.00001. (e) For right ventricular (RV) relative wall thickness (RWT), H1 *vs* H2 *vs* H3 was significant (*P* < 0.01) on three‐way ANOVA and H1 *vs* H3 was significant (*P* < 0.01) on two‐way ANOVA. AV, aortic valve; EDA, end‐diastolic area; IVS, interventricular septum; LV, left ventricular; MV, mitral valve; PV, pulmonary valve; RT, relative thickness; SI, sphericity index; TV, tricuspid valve.

**Figure 5:**
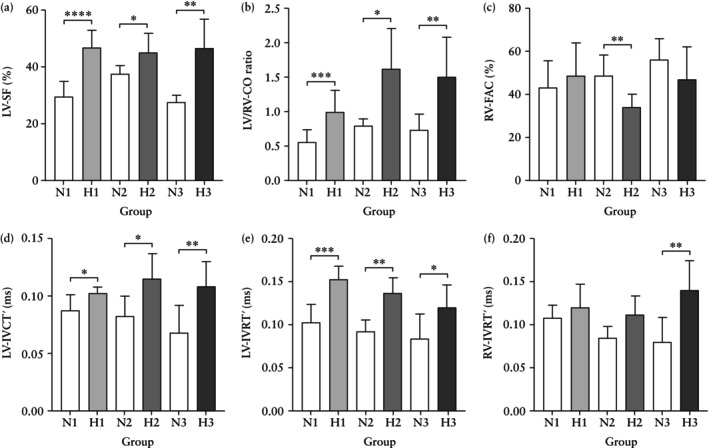
(a–f) Cardiac systolic and diastolic function in normoxic and chronically hypoxic sheep fetuses. Data are shown as column plots with error bars representing mean ± SD for alterations in cardiac function in chronically hypoxic sheep fetuses after 11 days (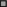, H1), after 17 days (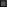, H2) and after 32 days (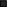, H3) of chronic hypoxia compared to gestational age‐matched normoxic controls (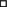, N1; N2; N3). Significant differences for comparison of normoxic *vs* hypoxic groups: *, *P* < 0.05; **, *P* < 0.01; ***, *P* < 0.001; ****, *P* < 0.00001. (c) For right ventricular (RV) fractional area change (FAC), H1 *vs* H2 *vs* H3 was significant (*P* < 0.01) on two‐way ANOVA. Myocardial time intervals were derived by pulsed‐wave tissue Doppler imaging and normalized by cardiac cycle length, adjusting for variation in heart rate. CO, cardiac output; IVCT′, isovolumetric contraction time; IVRT′, isovolumetric relaxation time; LV, left ventricular; SF, shortening fraction.

**Figure 6:**
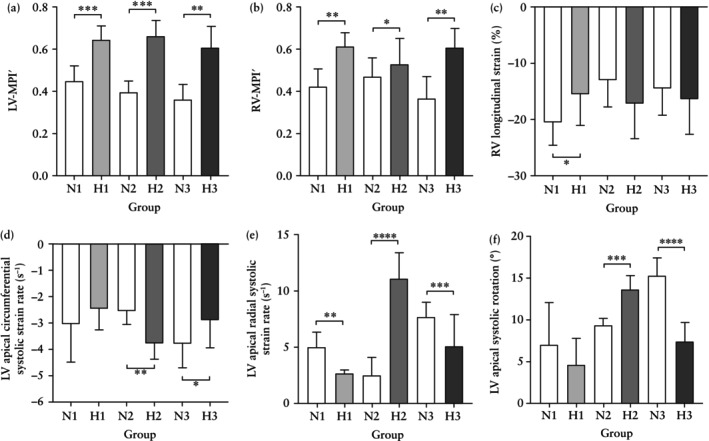
(a–f) Global myocardial performance and deformation in normoxic and chronically hypoxic sheep fetuses. Data are shown as column plots with error bars representing mean ± SD for alterations in global myocardial performance (a,b) and myocardial deformation (c–f) in chronically hypoxic sheep fetuses after 11 days (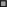, H1), after 17 days (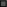, H2) and after 32 days (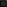, H3) of chronic hypoxia compared to gestational age‐matched normoxic controls (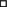, N1; N2; N3). Significant differences are for comparison of normoxic *vs* hypoxic groups: *, *P* < 0.05; **, *P* < 0.01; ***, *P* < 0.001; ****, *P* < 0.00001. Indices of myocardial deformation were derived by speckle‐tracking echocardiography. LV, left ventricular; MPI′, myocardial performance index derived by pulsed‐wave tissue Doppler imaging; RV, right ventricular.

Compared to the gestational age‐matched normoxic group (N1), fetuses after 11 days of chronic hypoxia (H1) showed the following alterations. With respect to cardiac geometry, H1 fetuses showed significantly reduced PV dimension, increased values for LV‐EDD, LV‐EDL and LV‐EDA, with elevated ratios for AV/PV dimension, MV/TV dimension and the LV/RV‐EDA ratio (Table 1, Figures 3 and 4). With respect to systolic function, H1 fetuses showed significantly reduced RV‐CO and RV‐ET′, increased LV‐SF and LV/RV‐CO ratio and a prolonged LV‐IVCT′ (Table 2, Figures 3 and 5). With respect to diastolic function, H1 fetuses showed a significant reduction in the IVS‐E′/A′ ratio and prolonged RV‐RT′ (Tables 2 and S1, Figures 3 and 5). With respect to global myocardial performance and deformation, H1 fetuses showed a significant increase in biventricular MPI′, a decrease in biventricular longitudinal myocardial strain, and a decrease in LV apical radial strain rate (Table 3, Figures 3 and 6).

**Table 1:** Effect of progressive hypoxia from 105 days of gestation (dGA) on fetal cardiac geometry in sheep, determined by echocardiography at three timepoints

	117 ± 1 dGA		124 ± 1 dGA		133 ± 3 dGA	
Characteristic	N1 (*n* = 13)	H1 (*n* = 5)	N2 (*n* = 5)	H2 (*n* = 6)	N3 (*n* = 7)	H3 (*n* = 6)
Left‐sided structures						
AV dimension (mm)	6.5 ± 0.7	6.9 ± 0.6	7.3 ± 0.3	7.7 ± 0.7	7.5 ± 0.6§	8.9 ± 1.0*,††
AV/PV dimension ratio	0.8 ± 0.1	1.0 ± 0.1*	0.9 ± 0.1	1.1 ± 0.1*	0.9 ± 0.1	1.1 ± 0.1*
MV dimension (mm)	6.9 ± 0.8	7.9 ± 1.2	7.6 ± 1.6	9.0 ± 1.3*	7.8 ± 0.5§	9.3 ± 1.2*
MV/TV dimension ratio	0.9 ± 0.1	1.2 ± 0.1*	0.9 ± 0.1	1.3 ± 0.1*	0.9 ± 0.0	1.1 ± 0.2*
LV‐EDD (mm)	10.9 ± 1.5	12.8 ± 1.3*	13.3 ± 1.3†	14.0 ± 1.8	12.8 ± 1.2§	15.3 ± 1.5*,††
LV/RV‐EDD ratio	1.1 ± 0.2	0.8 ± 0.3	1.2 ± 0.1	1.3 ± 0.1	0.9 ± 0.1‡	1.3 ± 0.2*
LV‐EDL (mm)	21.5 ± 2.7	24.6 ± 2.3*	26.8 ± 3.9†	25.7 ± 2.3	29.2 ± 1.9§	28.8 ± 3.7
LV‐SI	0.51 ± 0.09	0.52 ± 0.08	0.50 ± 0.05	0.54 ± 0.04	0.44 ± 0.04	0.54 ± 0.11*
LV‐EDA (mm^2^)	19.3 ± 4.6	25.8 ± 3.1*	26.8 ± 5.2†	31.3 ± 7.3	30.2 ± 4.3§	39.2 ± 6.2*,††
LV/RV‐EDA ratio	1.1 ± 0.2	1.3 ± 0.1*	1.2 ± 0.4	1.7 ± 0.5*	0.9 ± 0.1	1.4 ± 0.2*
LV‐RWT	0.55 ± 0.12	0.60 ± 0.06	0.46 ± 0.04	0.57 ± 0.09	0.50 ± 0.07	0.53 ± 0.07*,††
IVS‐RT	0.56 ± 0.14	0.63 ± 0.07	0.46 ± 0.04	0.60 ± 0.07*	0.50 ± 0.07	0.70 ± 0.09*
Right‐sided structures						
PV dimension (mm)	8.0 ± 0.5	7.0 ± 0.7*	8.1 ± 0.8	7.1 ± 0.7*	8.3 ± 0.7	8.1 ± 0.6**,††
TV dimension (mm)	7.7 ± 1.0	6.9 ± 1.0	8.9 ± 0.9†	7.3 ± 1.0*	8.8 ± 0.6§	8.3 ± 1.0††
RV‐EDD (mm)	10.5 ± 1.9	11.0 ± 3.0	9.2 ± 1.0	11.0 ± 1.5	14.3 ± 1.4‡,§	12.3 ± 1.4*
RV‐EDL (mm)	20.3 ± 3.0	20.6 ± 2.3	23.8 ± 1.3†	21.3 ± 1.4*	27.8 ± 2.7‡,§	25.8 ± 3.5**,††
RV‐SI	0.53 ± 0.11	0.54 ± 0.12	0.45 ± 0.05	0.52 ± 0.08	0.52 ± 0.04	0.49 ± 1.0
RV‐EDA (mm^2^)	18.4 ± 3.3	20.4 ± 3.0	23.8 ± 2.9†	18.7 ± 2.7*	33.5 ± 5.4‡,§	28.3 ± 6.2*,**,††
RV‐RWT	0.58 ± 0.11	0.56 ± 0.11	0.56 ± 0.05	0.74 ± 0.14*,¶	0.47 ± 0.05‡,§	0.74 ± 0.18*,††

Data are given as mean ± SD for normoxic fetuses (N) and hypoxic fetuses (H).

Significant differences (*P* < 0.05) are:

* , N1 *vs* H1 or N2 *vs* H2 or N3 *vs* H3;

† , N1 *vs* N2;

‡ , N2 *vs* N3;

§ , N1 *vs* N3;

¶ , H1 *vs* H2;

** , H2 *vs* H3;

†† , H1 *vs* H3.

AV, aortic valve; EDA, end‐diastolic area; EDD, end‐diastolic dimension; EDL, end‐diastolic length; IVS, interventricular septum; LV, left ventricular; MV, mitral valve; PV, pulmonary valve; RT, relative thickness; RV, right ventricular; RWT, relative wall thickness; SI, sphericity index; TV, tricuspid valve.

**Table 2:** Effect of progressive hypoxia from 105 days of gestation (dGA) on fetal systolic and diastolic function in sheep, determined by echocardiography at three timepoints

	117 ± 1 dGA		124 ± 1 dGA		133 ± 3 dGA	
Characteristic	N1 (*n* = 13)	H1 (*n* = 5)	N2 (*n* = 5)	H2 (*n* = 6)	N3 (*n* = 7)	H3 (*n* = 6)
Systolic function						
Fetal heart rate (bpm)	183 ± 21	190 ± 38	172 ± 28	175 ± 17	159 ± 8§	152 ± 10**,††
Left ventricle						
LV‐SF (%)	29.5 ± 5.3	46.7 ± 6.1*	37.6 ± 2.9†	43.9 ± 6.7*	27.3 ± 6.0‡	46.5 ± 10*
LV‐CO (mL/min)	436 ± 136	490 ± 157	611 ± 85†	854 ± 259¶	676 ± 239	1017 ± 419††
Combined CO (mL/min)	1236 ± 264	1006 ± 411	1395 ± 258	1397 ± 301	1616 ± 463§	1699 ± 488††
LV/RV‐CO ratio	0.55 ± 0.18	1.00 ± 0.31*	0.80 ± 0.10†	1.62 ± 0.59*	0.74 ± 0.23	1.50 ± 0.58*
LV‐ET′ (normalized) (ms)	0.426 ± 0.040	0.398 ± 0.037	0.433 ± 0.015	0.380 ± 0.022*	0.423 ± 0.022	0.386 ± 0.036
LV‐IVCT′ (normalized) (ms)	0.087 ± 0.022	0.103 ± 0.005*	0.083 ± 0.013	0.113 ± 0.021*	0.068 ± 0.022§	0.110 ± 0.020*
Right ventricle						
RV‐FAC (%)	43 ± 13	48 ± 15	49 ± 10	34 ± 6*,¶	56 ± 10§	47 ± 15
RV‐CO (mL/min)	800 ± 180	523 ± 253*	783 ± 182	542 ± 114*	940 ± 288	681 ± 148
RV‐ET′ (normalized) (ms)	0.447 ± 0.050	0.356 ± 0.043*	0.367 ± 0.052†	0.427 ± 0.066	0.489 ± 0.023‡	0.395 ± 0.039*
RV‐IVCT′ (normalized) (ms)	0.081 ± 0.018	0.095 ± 0.021	0.083 ± 0.022	0.107 ± 0.014	0.095 ± 0.026	0.096 ± 0.020
Diastolic function						
LV‐IVRT′ (normalized) (ms)	0.102 ± 0.020	0.151 ± 0.016*	0.089 ± 0.013	0.138 ± 0.018*	0.084 ± 0.026	0.120 ± 0.024*
RV‐IVRT′ (normalized) (ms)	0.105 ± 0.017	0.121 ± 0.025	0.087 ± 0.015	0.112 ± 0.021	0.082 ± 0.027	0.140 ± 0.033*
IVS‐E′/A′ ratio	0.64 ± 0.14	0.52 ± 0.11*	0.66 ± 0.06	0.70 ± 0.2¶	0.62 ± 0.10	0.67 ± 0.07††

Data are given as mean ± SD for normoxic fetuses (N) and hypoxic fetuses (H).

Significant differences (*P* < 0.05) are:

* , N1 *vs* H1 or N2 *vs* H2 or N3 *vs* H3;

† , N1 *vs* N2;

‡ , N2 *vs* N3;

§ , N1 *vs* N3;

¶ , H1 *vs* H2;

** , H2 *vs* H3;

†† , H1 *vs* H3.

′, cardiac indices derived by pulsed‐wave tissue Doppler imaging.

Myocardial intervals are normalized by cardiac cycle length adjusting for difference in fetal heart rate.

CO, cardiac output; E′/A′, early‐to‐late peak diastolic myocardial velocity ratio; ET′, ejection time; FAC, fractional area change; IVCT′, isovolumetric contraction time; IVRT′, isovolumetric relaxation time; IVS, interventricular septum; LV, left ventricular; RV, right ventricular; SF, shortening fraction.

**Table 3:** Effect of progressive hypoxia from 105 days of gestation (dGA) on fetal global myocardial performance and deformation in sheep, determined by echocardiography at three timepoints

	117 ± 1 dGA		124 ± 1 dGA		133 ± 3 dGA	
Characteristic	N1 (*n* = 13)	H1 (*n* = 5)	N2 (*n* = 5)	H2 (*n* = 6)	N3 (*n* = 7)	H3 (*n* = 6)
Global myocardial performance						
LV‐MPI′	0.45 ± 0.07	0.64 ± 0.07*	0.40 ± 0.05	0.66 ± 0.07*	0.36 ± 0.07§	0.60 ± 0.10*
MAPSE (mm)	7.4 ± 1.8	8.1 ± 0.9	9.7 ± 1.3†	9.9 ± 1.4¶	9.1 ± 1.5	11.8 ± 2.8*,††
SAPSE (mm)	6.5 ± 1.4	6.7 ± 0.7	8.4 ± 1.9†	7.3 ± 1.0	9.2 ± 2.2§	8.9 ± 0.8**,††
RV‐MPI′	0.42 ± 0.08	0.61 ± 0.07*	0.47 ± 0.09	0.53 ± 0.12*	0.36 ± 0.10	0.60 ± 0.10*
TAPSE (mm)	9.0 ± 2.2	9.5 ± 1.5	10.3 ± 1.1	9.6 ± 2.3	10.0 ± 2.4	11.2 ± 2.6
Myocardial deformation						
LV longitudinal systolic strain (%)	–17.5 ± 1.9	–14.4 ± 1.1*	–19.2 ± 3.6	–14.9 ± 2.5*	–19.5 ± 4.7	–19.5 ± 8.3
RV longitudinal systolic strain (%)	–20.5 ± 4.2	–15.5 ± 3.7*	–13.1 ± 4.7†	–17.1 ± 6.2	–14.5 ± 4.8§	–16.4 ± 3.6
LV basal circumferential systolic strain (%)	–13.7 ± 3.1	–15.7 ± 7.3	–15.0 ± 6.3	–11.6 ± 2.5	–9.4 ± 3.8§	–18.4 ± 4.4*,**
LV basal circumferential systolic strain rate (s^−1^)	–2.7 ± 0.7	–2.7 ± 1.0	–2.7 ± 1.4	–2.5 ± 0.8	–1.7 ± 0.7§	–3.2 ± 0.9*
LV apical circumferential systolic strain (%)	–14.6 ± 8.8	–14.7 ± 2.9	–15.0 ± 0.2	–18.6 ± 0.2*,¶	–16.9 ± 3.2	–18.6 ± 5.7
LV apical circumferential systolic strain rate (s^−1^)	–3.0 ± 1.6	–2.4 ± 0.8	–2.5 ± 0.6	–3.7 ± 0.6*,¶	–3.8 ± 0.9	–2.9 ± 1.1*
LV apical radial strain (%)	27.0 (26.0–28.0)	23.8 (21.0–25.5)	23.7 (22.0–24.5)	69.7 (66.2–79.2)*	50.6 (48.0–53.6)‡,§	28.6 (27.1–32.3)*,**
LV apical radial strain rate (s^−1^)	4.5 (4.3–4.6)	2.7 (2.6–2.8)*	2.5 (2.4–2.7)	11.8 (11.7–11.9)*,¶	7.6 (7.5–7.6)‡,§	4.8 (3.5–4.9)*,**
LV apical systolic rotation (°)	7.0 ± 4.8	4.8 ± 3.0	9.3 ± 0.8	13.6 ± 1.7*	15.3 ± 2.2‡,§	7.4 ± 2.3*,**
PSS present	4/13 (31)	4/5 (80)*	2/5 (40)	5/6 (83)*	4/7 (57)§	5/6 (83)*

Data are given as mean ± SD, median (interquartile range) or *n/N* (%) for normoxic fetuses (N) and hypoxic fetuses (H). Significant differences (*P* < 0.05) are:

* , N1 *vs* H1 or N2 *vs* H2 or N3 *vs* H3;

† , N1 *vs* N2;

‡ , N2 *vs* N3;

§ , N1 *vs* N3;

¶ , H1 *vs* H2;

** , H2 *vs* H3;

†† , H1 *vs* H3.

Post‐systolic shortening (PSS) was assessed from the left ventricular (LV) longitudinal strain curves derived from speckle‐tracking echocardiography.

′, cardiac indices derived by pulsed‐wave tissue Doppler imaging; MAPSE, mitral annular plane systolic excursion; MPI′, myocardial performance index; RV, right ventricular; SAPSE, septal annular plane systolic excursion; TAPSE, tricuspid annular plane systolic excursion.

### Cardiac alterations at 17 days of hypoxia

Compared to the gestational age‐matched normoxic group (N2), fetuses after 17 days of chronic hypoxia (H2) showed the following alterations. With respect to cardiac geometry, H2 fetuses showed significantly decreased PV and TV dimensions with increased AV/PV and MV/TV dimension ratios. In addition, H2 fetuses demonstrated significantly reduced RV‐EDL and RV‐EDA, and increased values for MV dimensions and the RV‐RWT and IVS relative thickness (Table 1, Figures 3 and 4).

With respect to systolic function, H2 fetuses showed a significant decrease in RV‐FAC, with persistently lower RV‐CO and a progressively increased LV‐SF and LV/RV‐CO ratio. These fetuses also showed a significant decrease in the LV‐ET′ and a persistent increase in the LV‐IVCT′ (Table 2, Figures 3 and 5). With respect to diastolic function, H2 fetuses showed significantly increased LV‐IVRT′ and decreased LV‐RT′ and RV‐RT′ (Tables 2 and S1, Figures 3 and 5).

With respect to global myocardial performance and deformation, H2 fetuses showed persistently elevated LV‐MPI′, lower LV longitudinal systolic strain but greater LV apical circumferential and radial strain and strain rate and greater LV apical systolic rotation (Table 3, Figures 3 and 6).

### Cardiac alterations at 32 days of hypoxia

Compared to the gestational age‐matched normoxic group (N3), fetuses after 32 days of hypoxia (H3) showed the following alterations. With respect to cardiac geometry, H3 fetuses showed significantly increased AV/PV dimension ratio, MV dimension, MV/TV dimension ratio and LV/RV‐EDA ratio. These fetuses also showed a significant increase in the AV dimension, LV‐ and RV‐RWT and IVS relative thickness, and reduced RV‐EDD but enlarged LV‐EDD. The latter geometric change resulted in a significantly greater LV/RV‐EDD ratio and a more globular‐shaped left ventricle, evident from an elevated LV‐SI (Table 1, Figures 3 and 4).

With respect to systolic function, H3 fetuses showed a significantly increased LV‐SF and LV/RV‐CO ratio. These fetuses also showed a significant reduction in RV‐ET′, while the LV‐IVCT′ interval remained prolonged (Table 2, Figures 3 and 5). With respect to diastolic function, H3 fetuses showed persistently prolonged LV‐IVRT′ and a significantly increased RV‐IVRT′ (Table 2, Figures 3 and 5). With respect to global myocardial performance and deformation, H3 fetuses showed an increase in both LV‐MPI′ and RV‐MPI′, greater MAPSE, LV basal circumferential strain and strain rate, but lower values for LV apical radial strain and strain rate and for LV apical systolic rotation (Table 3, Figures 3 and 6). Additionally, hypoxic fetuses demonstrated a significantly increased percentage of cases with PSS of LV longitudinal myocardial deformation at all three timepoints of assessment (Table 3).

### Gestational cardiac changes

Regarding cardiac geometry, both normoxic and chronically hypoxic fetuses showed growth‐dependent geometrical changes with advancing gestational age, reflected in increased valvular and biventricular dimensions (Tables 1 and S1). However, in contrast to fetuses of normoxic pregnancies that showed gestation‐dependent increases in RV‐EDD, LV‐EDL and a decreased LV/RV‐EDD, these changes did not reach significance with advancing gestation in all groups of chronically hypoxic fetuses. With advancing gestation, the RV‐RWT significantly decreased in normoxic fetuses from N1 to N3, whereas this parameter in chronically hypoxic fetuses increased significantly from H1 to H2 and then remained the same in H3. In contrast, the LV‐RWT in normoxic fetuses remained unchanged, while hypoxic fetuses demonstrated a significant decrease in LV‐RWT between the H1 and H3 groups (Table 1).

With respect to systolic function, both normoxic and chronically hypoxic fetuses showed a reduction in fetal heart rate with advancing gestational age, but this effect appeared more pronounced in chronically hypoxic fetuses (Table 2). There was a significant increase in LV‐CO between groups in chronically hypoxic fetuses that did not occur in normoxic fetuses. Conversely, with advancing gestation, normoxic fetuses demonstrated significant alterations in the RV‐ET′ (decreased between N1 and N2, and then increased between N2 and N3), an increase in RV‐FAC and a decrease in LV‐IVCT′ between N1 and N3 groups; all these changes did not occur in chronically hypoxic fetuses (Table 2).

With respect to diastolic function, there was an increase in the IVS‐E′/A′ ratio and a decrease in RV‐RT′ with advancing gestational age in chronically hypoxic fetuses that did not occur in normoxic fetuses, whereas the normoxic group showed gestation‐related increases in LV‐RT′ and RV‐RT′ between N1 and N2 that were not evident in hypoxic fetuses (Tables 2 and S1).

With respect to global myocardial performance and deformation in normoxic fetuses, there was a decrease in LV‐MPI′, RV longitudinal systolic strain and LV basal circumferential systolic strain and an increase in LV apical radial strain and LV apical systolic rotation at N3 compared with N1. The percentage of cases with PSS was also significantly increased at N3, which was not observed in the hypoxic group (Table 3). Furthermore, there was an increase in MAPSE and SAPSE with advancing gestation in both normoxic and chronically hypoxic fetuses (Table 3).

### Intra‐ and interobserver reproducibility

The limits of agreement and ICC showed good to excellent intra‐ and interobserver agreement for all fetal TDI and STE indices obtained in the same frame (ICC, 0.83–1.0). When STE measurements were repeated in a different frame, the intra‐ and interobserver agreement remained robust (ICC, 0.61–0.78) (Table S2).

## DISCUSSION

The data in this study support the hypothesis that varying durations of chronic hypoxia differentially affect cardiac structure, cardiac function and myocardial deformation in the late‐gestation fetus.

### Cardiac structure

Relative to normoxic fetuses, which exhibit RV dominance^3^, ^6^, hypoxic sheep fetuses showed a switch towards LV preponderance, evident from increases in the ratio of LV/RV‐EDA and in the dimension ratios of AV/PV and MV/TV, with a significant increase in the LV/RV‐EDD ratio and LV‐SI indicating a more globular and larger left ventricle by 33 dGA (Table 1, Figure 4). These changes may be explained by the fetal brain‐sparing response to hypoxia^3^. Cardiac adaptation to chronic preload in hypoxic fetuses is supported by LV elongation, widening and dilatation, leading to a dilated, globular‐shaped and hypertrophied LV phenotype, characteristic of eccentric hypertrophy^27^. Similarly, the cardiac adaptation to chronic afterload in hypoxic fetuses is supported by a decrease in RV chamber size and an increase in RV wall thickness, characteristic of concentric hypertrophy^27^, ^28^. These adaptive changes in fetal biventricular chamber shape and wall thickness are necessary, as they reduce wall stress and maintain CO during sustained hypoxia^29^. These data are consistent with histological findings in other studies reporting cardiac hypertrophy during chronic hypoxia in different animal models^1^, ^30^, ^31^, ^32^, ^33^, and with clinical studies reporting a dilated and globular hypertrophied left ventricle in the human FGR fetus^2^, ^23^, ^34^, ^35^, ^36^, ^37^, ^38^, ^39^.

### Cardiac function

Compared with normoxic fetuses, cardiac functional changes in the hypoxic fetuses in the present study complement the morphological data, revealing that hypoxic fetal sheep had a reduced RV output at 11 days and at 17 days of exposure, with a further decrease in RV‐ET′ by 32 days of hypoxia. In contrast, LV‐SF was elevated at all timepoints of assessment, while fetal heart rate and LV‐CO and combined CO remained similar between normoxic and hypoxic groups. The result was a doubling of the LV output across all durations of hypoxia (Table 2, Figure 5), also supporting a shift towards LV dominance in hypoxic fetuses because of fetal brain‐sparing^23^, ^40^. According to the Frank–Starling mechanism, a decrease in RV preload will reduce RV contractility, whereas an increase in LV volume load will favor an increase in SF. Accordingly, in human FGR pregnancies, fetuses show a reduced fetal RV output, while LV or combined CO remain unchanged^23^, ^41^. Interestingly, the left ventricle compared to the right ventricle has higher sensitivity to worsening hypoxia^42^. In agreement, data in the present study show that hypoxic sheep fetuses revealed prolonged LV‐IVCT′ across all durations of hypoxia, evident after 11 days of exposure. In contrast, RV‐IVCT′ remained unaffected, probably owing to the reduced RV preload across all durations of hypoxia. In the present study, hypoxic fetuses showed biventricular diastolic dysfunction, evident from a decrease in IVS‐E′/A′ at 11 days of hypoxia and a prolongation of LV‐ and RV‐IVRT′. The LV diastolic impairment presented after 11 days, preceding RV compromise after 32 days, further supporting greater LV susceptibility to worsening hypoxia^42^. Importantly, maternal hypoxia may be affecting the fetal heart directly and indirectly through worsening of placental function. We have reported previously that this level of maternal hypoxia may impact placental function, increase uteroplacental vascular resistance and reduce fetal growth in sheep pregnancy^43^. The latter study of Tong *et al*.^43^ and other reports also refer to fetal brain‐sparing measured by both Doppler blood flow waveform changes and measurement *in vivo* using Transonic flow probes implanted around the fetal carotid and femoral arteries^5^, ^43^. We have also isolated direct effects of chronic hypoxia on cardiac structure and function using the chicken embryo model, thereby independent of the effects of hypoxia on the mother and/or placenta^44^.

### Myocardial performance and deformation

Our data showed an increase in biventricular MPI′ in all hypoxic fetal sheep groups, at the expense of prolonged LV‐IVCT′, LV‐IVRT′ and RV‐IVRT′ intervals and reduced RV‐ET′ (Tables 2 and 3, Figures 5 and 6). Remarkably, most sheep fetuses during all durations of hypoxia revealed LV dyssynchrony, evidenced by the high percentage of cases with PSS, concordant with findings in human FGR fetuses^20^. In addition, there was a significant decrease in LV and RV longitudinal systolic strain after 11 days, but the decrease only persisted in LV longitudinal systolic strain after 17 days of hypoxia. While apical, circumferential, radial and rotational myocardial deformation indices intensified in hypoxic fetuses at 17 days, values for these variables were lower after 32 days of chronic hypoxia. The reduced biventricular longitudinal myocardial deformation observed after 11 days of hypoxia may reflect the earliest signs of myocardial damage, because the longitudinal fibers that line the endocardium are furthest from coronary perfusion^45^. In contrast, LV circumferential fibers are better at responding to afterload^46^, and this may be reflected in the elevated circumferential and radial myocardial deformation indices in hypoxic fetal sheep in the present study. The transient elevation of circumferential, radial and rotational myocardial deformation at 17 days of hypoxic exposure in fetal sheep may represent cardiac compensatory mechanisms for improving LV diastolic dysfunction under increased LV preload^47^, ^48^ (Table 3, Figure 6). Similar cardiac changes have been reported as an early sign of subendocardial (longitudinal fibers) impairment in experimental animal and human studies^48^, ^49^, ^50^, ^51^. The alteration in LV longitudinal strain at 32 days of hypoxia could be due to the decrease in apical radial strain because longitudinal and circumferential myocardial fibers work in ‘agonist–antagonist’ fashion and are influenced by changes in loading conditions at term^52^. The switch from an increase in apical, radial and rotational myocardial strain and strain rate after 17 days to a marked reduction in these values after 32 days of hypoxia suggests that compensatory cardiac reserves became exhausted, signaling decompensation and hemodynamic compromise. This association between reduced rotational mechanics with increased afterload and LV sphericity has been reported in the human FGR fetus^26^, ^53^, ^54^, ^55^.

### Gestational cardiac changes in normoxic fetuses

The significant changes in the normoxic groups in the last weeks of pregnancy reflect gestation‐related adaptations to fetal growth, increased circulating volume and alteration in loading patterns due to maturational changes in the fetoplacental circulation^25^. An increased volume load of the right ventricle and reduced preload to the left ventricle could explain the findings in the control fetal group at term (N3): namely, increased RV‐EDD, RV‐FAC and RV‐ET′, whereas the left ventricle had reduced LV‐SF, improved LV intrinsic function (decreased LV‐IVCT′) and decreased basal circumferential strain. The reduction in RV longitudinal strain towards term may reflect the increased RV afterload. Our cardiac findings in the normoxic fetal sheep are consistent with measurements of human fetal cardiac adaptation to advancing gestation^19^, ^25^.

### Strengths and limitations

This is the first echocardiographic study to combine advanced imaging with a bespoke hypoxic chamber system to explore the fetal cardiac adaptation to progressive hypoxia in sheep, a species of high human translational value. The same fetal cardiologist (O.V.P.) conducted the echocardiography in all sheep fetuses in this study, and in human FGR fetuses in other clinical studies, using the same ultrasound machine, vendor‐specific STE software and protocol^19^, ^23^, ^24^, ^25^, with good intra‐ and interobserver repeatability^56^. A limitation of this study is that we could not adjust some cardiac parameters by fetal size, as fetal weight was not recorded at the end of the different periods of hypoxic exposure because, while most animals had an echocardiographic assessment at all three timepoints, some had the procedure at two timepoints only. However, we have reported that this ovine model of chronic hypoxia consistently promotes FGR^11^, ^43^, ^57^. Although associations between gender and fetal cardiac outcomes were not found, mixing of animal sexes could have increased variability, as FGR has been shown to induce sex‐specific effects^58^.

### Conclusions

The cardiac responses of fetal sheep in hypoxic pregnancy are similar to those in human FGR pregnancy, indicative of a primary role for chronic fetal hypoxia inducing fetal cardiac remodeling and dysfunction in human FGR pregnancy. Therefore, the application of advanced ultrasound techniques to the fetal heart may improve clinical diagnosis of the extent of fetal compromise to chronic hypoxia in human FGR pregnancy and expedite therapy to mitigate its impact.

## Supporting information


**Table S1** Effect of progressive hypoxia on additional fetal cardiac geometric and functional parameters in sheep, determined by echocardiography
**Table S2** Intra‐ and interobserver repeatability of fetal cardiac parameters in sheep

## Data Availability

The data that support the findings of this study are available on request from the corresponding author. The data are not publicly available due to privacy or ethical restrictions.
