# Supplementary material for: Translatable in‐vivo investigation of effects of progressive hypoxia in pregnancy on fetal cardiac structure and function in sheep
Source: Ultrasound Obstet Gynecol. 2025 Aug 18;66(4):486–98. doi: 10.1002/uog.29306 (PMC12488199; doi:10.1002/uog.29306)
Supplement: Supplementary file 1 — Table S1 Effect of progressive hypoxia on additional fetal cardiac geometric and functional parameters in sheep, determined by echocardiography Table S2 Intra‐ and interobserver repeatability of fetal cardiac parameters in sheep [file UOG-66-486-s001.docx]

**TRANSLATABLE *IN VIVO* INVESTIGATION OF PROGRESSIVE HYPOXIC PREGNANCY ON FETAL CARDIAC STRUCTURE AND FUNCTION IN SHEEP**

OV Patey, KL Botting, Y Niu, L Zhang, J Ma, SG Ford, W Tong, CM Coutinho, B Thilaganathan & DA Giussani

**SUPPLEMENTARY DATA**

**Supplemental Tables**

**Table S1.** Effect of progressive hypoxic pregnancy on additional fetal cardiac geometric and functional parameters determined by echocardiography

**Table S2.** Intra- and interobserver repeatability of fetal cardiac parameters in sheep

**Table S1. Effect of progressive hypoxic pregnancy on additional fetal cardiac function parameters determined by echocardiography**

| **Characteristics** | **Abbreviations** | **Groups** | | | | | |
| --- | --- | --- | --- | --- | --- | --- | --- |
|  |  | **1^st^ time point** | | **2^nd^ time point** | | **3^rd^ time point** | |
|  |  | **N1(n=13)** | **H1 (n=5)** | **N2 (n=5)** | **H2 (n=6)** | **N3 (n=7)** | **H3 (n=6)** |
| **CARDIAC GEOMETRY** |  | | | | | | |
| LV wall thickness, mm  (raw values) | LV WT | 2.9±0.5 | 3.6±0.6* | 3.1±0.5 | 3.7±0.5* | 3.2±0.4 | 3.8±0.8* |
| RV wall thickness, mm  (raw values) | RV WT | 3.0±0.4 | 3.0±0.3 | 3.2±0.3 | 4.0±0.6§ | 3.3±0.5 | 4.5±0.9§#* |
| IVS thickness, mm  (raw values) | IVS thickness | 3.0±0.6 | 4.0±0.1* | 3.2±0.4 | 4.2±0.4* | 3.2±0.4 | 5.3±0.5§#* |
| **SYSTOLIC FUNCTION** |  |  |  |  |  |  |  |
| **Left Ventricle:** |  |  |  |  |  |  |  |
| Aortic valve peak systolic velocities, cm/s | AV Vmax | 65.9±12.2 | 67.5±10.7 | 76.3±5.7 | 89.7±15.4*§ | 75.0±20.1 | 84.2±22.5 |
| Aortic valve velocity time integral, cm | AV VTI | 7.2 ±1.7 | 6.9±1.6 | 8.7±0.6 | 10.3±2.0*§ | 9.5±2.6‡ | 10.5±3.1# |
| Aortic valve velocity time integral, cm | AV VTI | 7.2 ±1.7 | 6.9±1.6 | 8.7±0.6 | 10.3±2.0*§ | 9.5±2.6‡ | 10.5±3.1# |
| Aortic to pulmonary valve velocity time integral ratio | AV/PV VTI | 0.83±0.17 | 0.97±0.08 | 1.03±0.28 | 1.32±0.38 | 0.93±0.33 | 1.27±0.32* |
| LV myocardial systolic velocity S’, cm/s | LV S’ | 7.2±2.4 | 6.9±1.3 | 7.0±2.9 | 8.4±2.1§ | 8.6±1.9 | 6.8±0.5§ |
| LV ejection time’, ms  (raw values) | LV ET’ | 144±10 | 138±10* | 150±9 | 134±5* | 160±31 | 151±14 |
| LV isovolumetric contraction time’, ms (raw values) | LV IVCT’ | 30±5 | 36±7* | 29±5 | 40±6* | 25±6‡ | 43±9* |
| IVS myocardial systolic velocity S’, cm/s | IVS S’ | 6.1±2.1 | 6.9±2.3 | 7.3±5.7 | 6.4±1.7 | 6.6±1.2 | 6.6±1.2 |
| **Right Ventricle:** |  |  |  |  |  |  |  |
| Pulmonary valve velocity time integral, cm | PV VTI | 8.7±1.5 | 7.7±1.6 | 8.8±1.9 | 8.1±2.3 | 10.6±2.0‡ | 8.4±2.3* |
| RV myocardial systolic velocity S’, cm/s | RV S’ | 7.3±2.1 | 7.3±1.2 | 8.5±2.6 | 6.7±1.5 | 7.5±4.3 | 8.2±2.1 |
| RV ejection time’, ms  (raw values) | RV ET’ | 150±15 | 130±23* | 138±27 | 154±20 | 172±8† | 152±14* |
| RV isovolumetric contraction time’, ms (raw values) | RV IVCT’ | 27±5 | 35±11 | 31±6 | 39±4 | 34±10 | 37±7 |
| **DIASTOLIC FUNCTION** | | | | | | | |
| **Left Ventricle:** |  |  |  |  |  |  |  |
| LV transvalvar early diastolic velocities, cm/s | LV E | 39±7 | 28±6* | 23±7† | 32±2 | 33±7 | 33±4 |
| LV transvalvar late diastolic velocities, cm/s | RV A | 48±13 | 43±9 | 36±9 | 49±7 | 46±11 | 45±4 |
| LV transvalvar early to late diastolic velocity ratio | LV E/A | 0.88±0.37 | 0.66±0.05 | 0.61±0.09 | 0.65±0.07 | 0.71±0.06 | 0.74±0.03 |
| LV myocardial early diastolic velocities, cm/s | LV E’ | 5.4±2.0 | 6.7±1.0 | 6.4±2.4 | 5.8±1.0 | 6.4±1.9 | 6.8±2.4 |
| LV myocardial late diastolic velocities, cm/s | LV A’ | 9.6±2.5 | 11.8±2.1 | 11.2±6.6 | 11.8±1.15 | 9.7±3.0 | 8.9±3.7 |
| LV myocardial early to late diastolic velocity ratio | LV E’/A’ | 0.57±0.13 | 0.58±0.16 | 0.66±0.24 | 0.49±0.06 | 0.67±0.10 | 0.59±0.08§ |
| LV transvalvar to myocardial early diastolic velocity ratio | LV E/E’ | 7.1±2.4 | 4.6±1.2 | 3.5±2.0† | 5.2±0.9 | 6.0±3.0 | 4.4±1.3 |
| Mitral valve velocity-time integral, mm | MV VTI | 5.5±0.9 | 5.3±1.8 | 3.1±0.9 | 6.1±1.0* | 5.4±1.0 | 6.0±1.5 |
| Mitral to tricuspid valve velocity-time integral ratio | MV/TV VTI | 0.77±0.39 | 1.20±0.39* | 0.76±0.15 | 1.07±0.28 | 0.91±0.18 | 0.86±0.06 |
| LV relaxation time’, ms  (raw values) | LV RT’ | 131±29 | 128±35 | 151±15† | 136±24* | 160±44 | 155±19 |
| LV relaxation time’, ms (normalised by cc) | LV RT’ normalised | 0.381±0.052 | 0.361±0.044 | 0.438±0.041† | 0.383±0.044* | 0.416±0.034 | 0.396±0.048 |
| LV isovolumetric relaxation time’, ms (raw values) | LV IVRT’ | 34±6 | 53±8* | 31±4 | 49±6* | 32±10 | 47±9*# |
| **Right Ventricle:** |  |  |  |  |  |  |  |
| RV transvalvar early diastolic velocities, cm/s | RV E | 40±6 | 24±3* | 38±2 | 38±5§ | 37±11 | 37±6 |
| RV transvalvar late diastolic velocities, cm/s | RV A | 53±13 | 38±7 | 46±17 | 53±11 | 45±7 | 48±13 |
| RV transvalvar early to late diastolic velocity ratio | RV E/A | 0.77±0.12 | 0.47±0.14* | 0.32±0.07† | 0.71±0.05 | 0.83±0.14 | 0.80±0.11 |
| RV myocardial early diastolic velocities, cm/s | RV E’ | 8.7±4.8 | 6.1±1.4 | 5.2±2.2 | 6.0±2.2 | 7.0±3.1 | 7.2±2.7 |
| RV myocardial late diastolic velocities, cm/s | RV A’ | 12.4±4.1 | 10.5±2.5 | 9.3±3.7 | 10.0±3.6 | 6.5±2.6 | 10.9±3.0 |
| RV myocardial early to late diastolic velocity ratio | RV E’/A’ | 0.71±0.24 | 0.59±0.14 | 0.56±0.05 | 0.61±0.15 | 0.74±0.15 | 0.65±0.09 |
| RV transvalvar to myocardial early diastolic velocity ratio | RV E/E’ | 5.6 (3.6) | 2.9 (1.3) | 1.3 (0.4) | 4.4 (7.6) | 5.5 (7.9) | 1.7 (4.7) |
| Tricuspid valve velocity-time integral, mm | TV VTI | 6.4±1.8 | 4.0±0.6* | 4.8±0.4 | 6.0±1.4 | 6.5±1.1 | 7.0±1.7 |
| RV relaxation time’, ms  (raw values) | RV RT’ | 35±6 | 44±8* | 33±3 | 41±8* | 29±11 | 54±13*# |
| RV relaxation time’, ms (normalised by cc) | RV RT’  normalised | 0.377±0.050 | 0.436±0.044* | 0.446±0.036† | 0.344±0.047*§ | 0.418±0.107 | 0.368±0.044# |
| RV isovolumetric relaxation time’, ms (raw values) | RV IVRT’ | 35±6 | 44±8 | 33±3 | 41±8 | 29±11 | 54±12* |
| **Interventricular Septum:** |  |  |  |  |  |  |  |
| IVS myocardial early diastolic velocities, cm/s | IVS E’ | 5.5±2.0 | 4.5±1.9 | 4.8±1.5 | 6.3±4.2 | 5.3±1.1 | 5.8±1.4 |
| IVS myocardial late diastolic velocities, cm/s | IVS A’ | 8.8±3.2 | 8.7±1.8 | 7.1±1.8 | 8.5±3.8 | 8.8±2.5 | 8.7±2.1 |
| IVS myocardial early to late diastolic velocity ratio | IVS E’/A’ | 0.64±0.14 | 0.52±0.11 | 0.66±0.06 | 0.70±0.20 | 0.62±0.11 | 0.67±0.07 |

Values are mean ± SD for normoxic fetuses (N) and hypoxic fetuses (H). Significant difference (P<0.05) are: *N1 *vs.* H1, N2 *vs.* H2, N3 *vs*. H3; †N1 *vs.* N2 *vs.* N3 groups; ‡N1 *vs.* N3 groups; §H1 *vs.* H2 *vs.* H3 groups; #H1 vs. H3 groups. IVS, interventricular septum; LV, left ventricular; ms, milliseconds; RV, right ventricular.

**Table S2. Intra- and inter-observer repeatability of fetal cardiac parameters in sheep**

| **Measurements** | **Fetal** | |
| --- | --- | --- |
|  | **ICC>0.7** | **LoA, p>0.05** |
| *Intra-observer measurement error (same frame), N=10* | | |
| All measurements | 34/35 (97%) | 34/35 (97%) |
| Tissue Doppler imaging | 17/17 (100%) | 17/17 (100%) |
| Speckle tracking imaging | 17/18 (94%) | 17/18 (94%) |
| *Intra-observer overall error (different frame), N=10* | | |
| All measurements | 30/35 (86%) | 32/35 (91%) |
| Tissue Doppler imaging | 16/17 (94%) | 17/17 (100%) |
| Speckle tracking imaging | 14/18 (78%) | 15/18 (89%) |
| *Inter-observer measurement error (same frame), N=10* | | |
| All measurements | 32/35 (91%) | 33/35 (94%) |
| Tissue Doppler imaging | 17/17 (100%) | 17/17 (100%) |
| Speckle tracking imaging | 15/18 (83%) | 16/18 (89%) |
| *Inter-observer overall error (different frame), N=10* | | |
| All measurements | 26/35 (74%) | 31/35 (89%) |
| Tissue Doppler imaging | 15/17 (88%) | 16/17 (94%) |
| Speckle tracking imaging | 11/18 (61%) | 15/18 (83%) |

Data are presented as n/N (%). *N*, number of fetuses; ICC, intra-class correlation coefficient;

LoA, limits of agreement (Pitman’s test).
